# Supplementary material for: A Versatile Three Dimensional Traction Force Microscopy Framework for Uncovering the Mechanics of Bio‐Adhesion
Source: Adv Sci (Weinh). 2025 Dec 17;13(10):e15497. doi: 10.1002/advs.202515497 (PMC12915117; doi:10.1002/advs.202515497)
Supplement: Supplementary file 1 — Supporting Information [file ADVS-13-e15497-s001.docx]

# Supplementary Materials

## S.1 Substrates manufacturing

The PDMS substrates were manufactured via spin coating process described in Table S1.

Table S1. The spin coating process for manufacturing PDMS substrate

| **Step** | **Materials** | **Coating speed (rpm)** | **Coating time (s)** | **Thickness (µm)** |
| --- | --- | --- | --- | --- |
| 1 | PDMS | 500 | 30 | 125 |
| 2 | PDMS | 500 | 30 | 125 |
| 3 | Particle/PDMS | 2100 | 260 | 15 |
| 4 | Pigment/PDMS | 2100 | 260 | 15 |

The formulation of the monomer and curing agent was 10:1 wt%. The PDMS layers were coated separately on an acrylic substrate via a spin coater (SPIN150i, POLOS, Germany). The coating speed (500 rpm) and coating time (30 s) were chosen for manufacturing the pure PDMS substrate with a thickness of approximately 200 – 300 µm. The third layer was made of PDMS with white micro particles and was spin-coated under 2100 rpm for 260 s. The fourth layer was spin coated with PDMS which was dyed with black silicone pigment (Easy Composites, UK) with 1 wt% under the same coating speed and time as the third layer. The cross-section of the substrate was examined by a microscope (Leica DMI4000B, Leica Microsystems, Wetzler, Germany).

## S.2 The tensile property of the PDMS substrate

The tension test on the particle-pigmented PDMS was conducted with the tensile strain up to about 25%. The maximum strain in our studies (accuracy validation and case study) was up to 13%. The strain-stress curves between the pure PDMS and the particle-pigmented PDMS are close, and almost identical in the strain range of 0-13%, shown in Figure S1. This indicates that the addition of particles and pigment has negligible effect on the mechanical performance of the PDMS.





Figure S1. The stress-strain curves of pure PDMS and particle-pigmented PDMS

## S.3 Experimental Determination of the Poisson’s Ratio of PDMS Substrate

To experimentally validate the Poisson’s ratio used in the finite-element reconstruction, quasi-static uniaxial tensile tests were conducted on thin PDMS films directly cut from the same speckled substrate used in the traction experiments, including both the ZnS:Cu particle and black pigment layers. The tensile specimens were cut from the PDMS substrate using a hydraulic press (SEALEY, UK) and a precision metal mould conforming to the ASTM D638 Type V geometry.

Quasi-static tests were conducted using a screw-driven load frame (Instron 5980, 1 kN load cell) at 20 °C and a displacement rate of 0.1 mm s⁻¹, corresponding to a nominal strain rate of approximately 0.01 s⁻¹. Each specimen surface was spray-coated to create a random speckle pattern suitable for DIC analysis, as shown in Figure S2a.


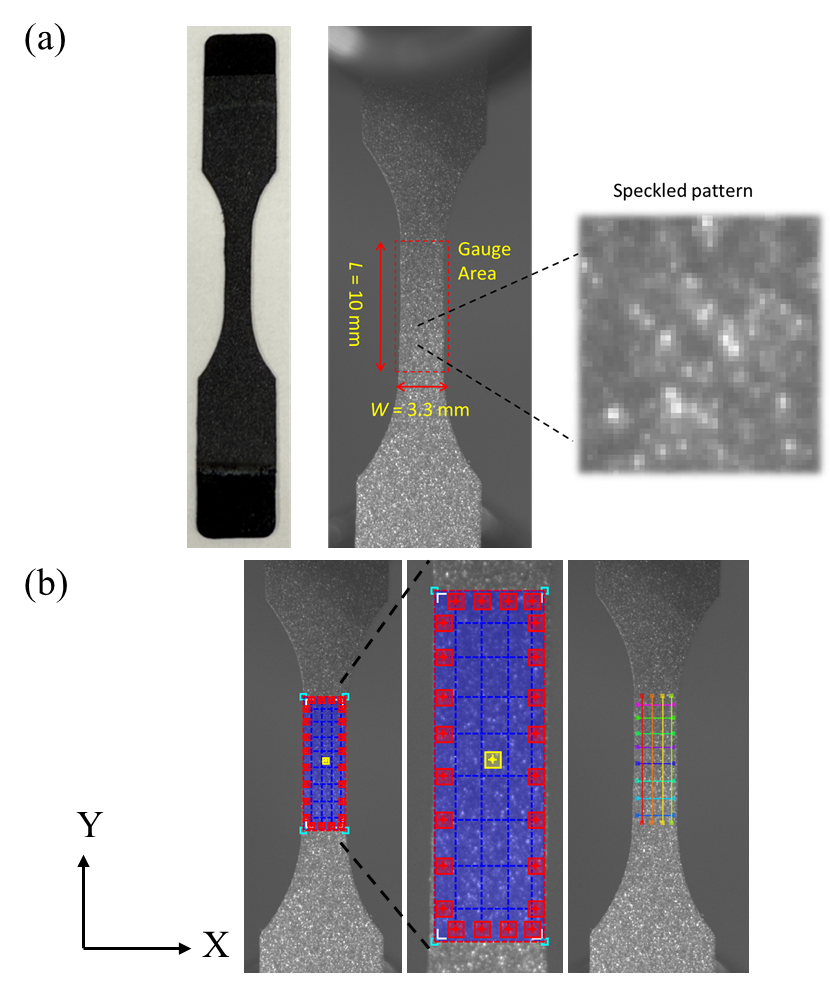


Figure S2. (a) Dog-bone specimen cut from the PDMS substrate for tensile testing, showing the applied random speckle pattern on the specimen surface; (b) Virtual strain gauges positioned along the longitudinal and transverse directions within the gauge section for DIC strain analysis.

Full-field strain fields were recorded using a Point Grey USB 2.0 CCD camera equipped with a Nikon 105 mm lens. The DIC analysis was performed in MatchID (version 2024.1.1) using an affine subset shape function and a zero-normalized sum of squared differences (ZNSSD) correlation criterion. The experimental and DIC parameters are summarized in Table S2. The strain window was set to 113 pixels, which is approximately equal to the width of the gauge section, allowing for a smoother strain field and to minimize noise.

Table S2. The DIC parameters for Poisson’s ratios measurement

| Image resolution (pixel) | 576 × 2048 |
| --- | --- |
| Field of view (pixel) | 121 × 1019 |
| Pixel-to-mm conversion | 0.024 |
| Speckle size (pixel) | 5-8 |
| Subset size (pixel) | 17 |
| Step size (pixel) | 4 |
| Strain tensor | Hencky (True) strain tensor |
| Strain window (pixel) | 57 × 57, Quadratic Quadrilateral |

The transverse ($\varepsilon_{transverse}$) and longitudinal ($\varepsilon_{longitudinal}$) true strains were extracted from the gauge section using virtual strain gauge (Figure S2b), and the Poisson’s ratio was computed up to an engineering strain of 1.5 according to

$$v=-\frac{\varepsilon_{transverse}}{\varepsilon_{longitudinal}}$$

The evolution of transverse and longitudinal true strains, together with the corresponding Poisson’s ratio, is shown in Figure S3. The measured Poisson’s ratio within the gauge section, excluding edge regions that may be affected by noise, was ν=0.45 ± 0.03. The selected value of ν=0.45 used in the finite-element reconstruction was further validated by comparing the total reconstructed traction force with the known gravitational force of a reference steel ball described in Section 2.3, demonstrating quantitative agreement within experimental uncertainty. This confirms that the adopted Poisson’s ratio accurately represents the mechanical response of the PDMS substrate used in this study.


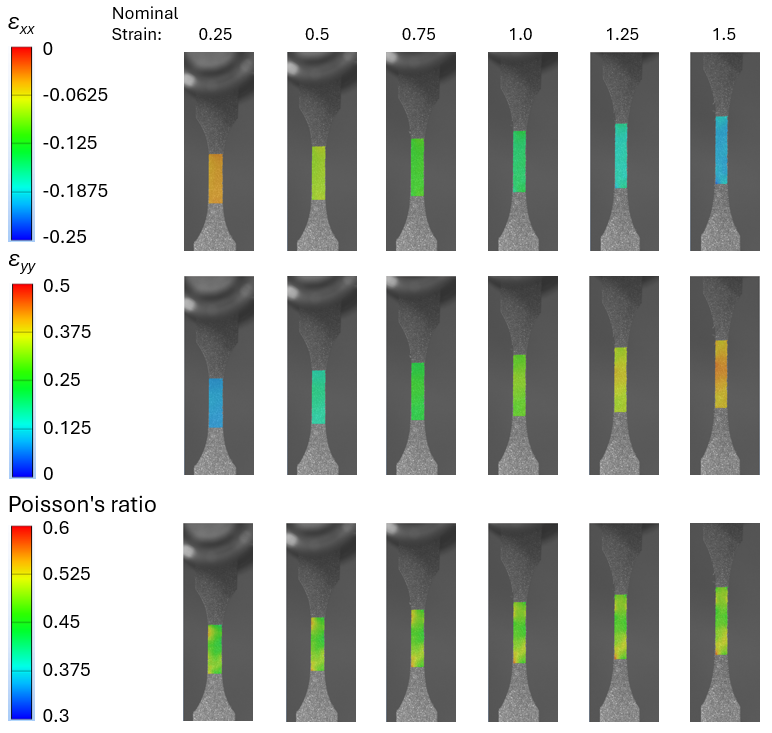


Figure S3. Evolution of true strain in the transverse (ε_xx_) and longitudinal (ε_yy_) directions, together with the corresponding Poisson’s ratio, as a function of nominal strain up to 1.5.

## S.4 The contact angle and bonding strength of the substrate

The contact angle was measured using DSA100 (KRUSS, Germany) to investigate the wettability of pristine PDMS and the speckled substrate. Droplets of deionized water were randomly dropped on the surface of the materials and the contact angle between the droplets and the materials were analysed as shown in Figure S4. Five measurements were conducted for each material. The contact angles for pristine PDMS and speckled substrate are 109.2° ± 4.7° and 110.2° ± 6.1°, respectively, showing no significant difference in macroscopic wettability between the two surfaces. This indicates that the addition of speckle particles and black pigment did not substantially alter the surface energy of PDMS.


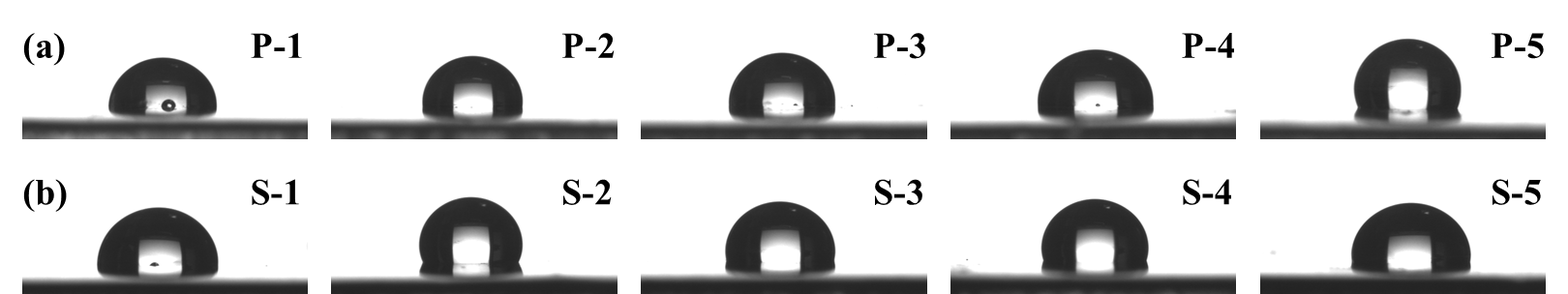


Figure S4. Contact angle images of (a) pristine PDMS samples (P-1 to P-5) and (b) speckled PDMS samples (S-1 to S-5).

To ensure reproducibility and isolate the effect of the surface modification, adhesion performance was compared using a standard 180° peeling test (ASTM D3330) with a commercial bonding tape (3M GPT-020F, RS, UK) rather than mussel plaques, since biological adhesion strength can vary considerably with mussel size, secretion activity, and environmental history (*1*). Tape segments with a total length of 300 ± 1 mm and a width of 25 ± 0.1 mm were bonded to the top surface of the PDMS substrates. As described in Section 2.1, the substrates consisted of either pristine or speckled PDMS mounted on an acrylic panel. The bonded length of each specimen was approximately 65 ± 2 mm. Peeling tests were performed using an Instron 68TM-10 testing machine at a loading rate of 5 mm/s. One grip clamped a folded 12 mm section at one end of the tape, while the other grip held the PDMS substrate. The experimental setup is illustrated in Figure S5a. Each test was repeated three times for both substrate types, and the results are summarised in Figure S5b.


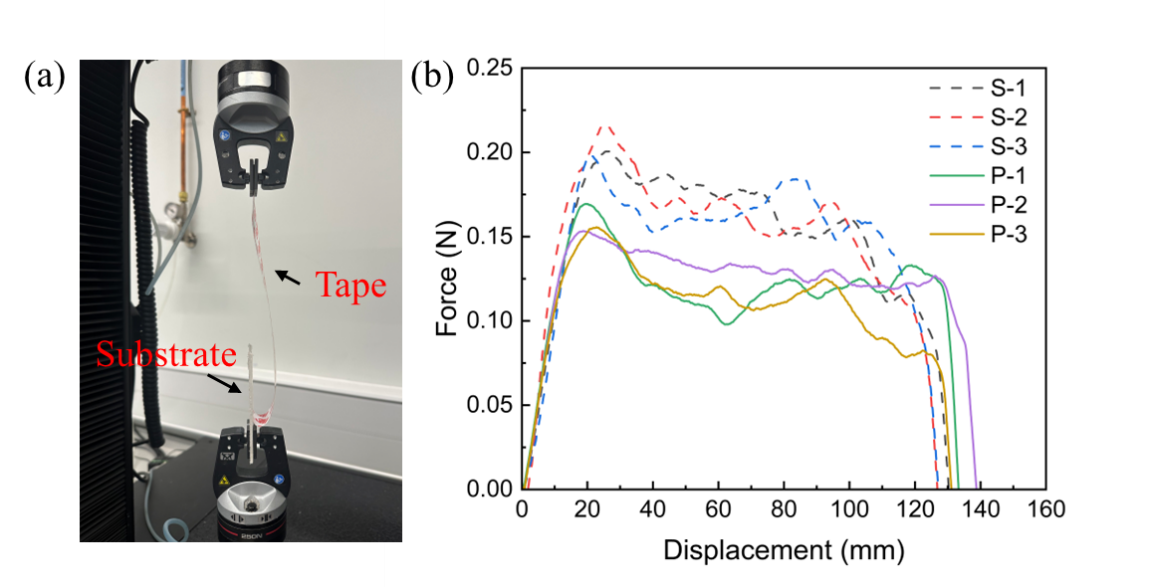


Figure S5. (a) Experimental setup for the 180° peeling test; (b) Load-displacement curves for tapes peeling from pristine PDMS substrate (P-1, P-2 and P-3) and speckled PDMS substrates (S-1, S-2 and S-3).

The peeling tests revealed that the speckled PDMS exhibited a higher steady-state peeling force of 0.175 N ± 0.015 N compared to 0.125 ± 0.018 N for pristine PDMS, corresponding to energy release rates of approximately 7.0 ± 0.6 J m⁻² and 5.0 ± 0.7 J m⁻², respectively. The displacement curves of the speckled PDMS displayed more pronounced fluctuations and earlier failure initiation, suggesting a heterogeneous interfacial structure. These results imply that while the surface chemistry remained largely unchanged, the introduction of pigment particles modified the microscale topography and local mechanical properties, leading to enhanced mechanical interlocking and crack-path deflection that increased overall adhesion but reduced interfacial uniformity and stability during peeling.

## S.5 Validation of Winkler foundation via FE simulation

FE simulations were conducted using a 3D elastic substate illustrated in Figure S6a (Young’s modulus E = 1.7 MPa, Poisson’s ratio ν = 0.30–0.45, thickness h = 0.3 mm) with a total surface area of 22.5 × 22.5 mm^2^. The central region (7.5 × 7.5 mm^2^) was subjected to uniform surface pressures in the z direction ranging from 0.05 and 0.25 MPa. The element type was C3D8R in ABAQUS notation, and the mesh density was approximately 475 elements/mm³, consistent with the DIC resolution. The nodes at the bottom surface were fully constrained to prevent any translational or rotational movement.


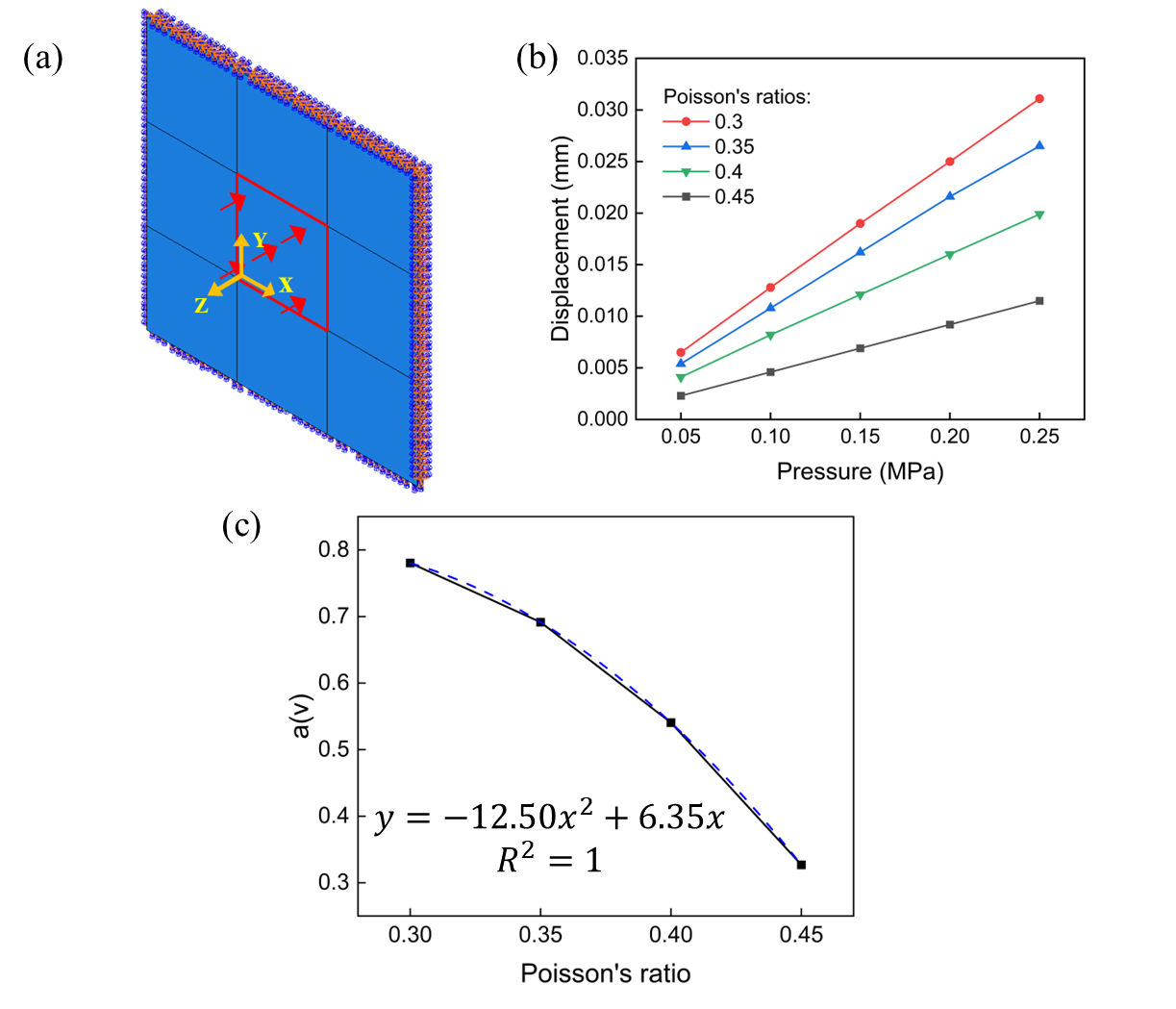


Figure S6. (a) The FE model used for validation of the Winkler foundation approximation;(b) The displacement-pressure curves of the substate with Poisson’s ratios varying from 0.30 to 0.45; (c) Fitting curve showing the relationship between the modifying parameter *a(ν)* and Poisson’s ratio

The resulting surface displacements varied linearly with applied pressure, consistent with linear-elastic response, see Figure S6b. The effective stiffness values obtained from the simulations, $k_{FE}=\frac{\sigma}{\delta_{z}}$, for different Poisson’s ratios are summarised in Table S3. The corresponding stiffness values calculated using Timoshenko solution, $k_{T}=\frac{E}{h}(1-v^{2})$ are also included for comparison. The results show that both the *k_FE_* and the *k_T_* increase with Poisson’s ratio. However, *k_FE_* exhibits more significant dependency, increasing by up to 172%, compared to only 14% for *k_T_*.

Table S3. Effective stiffness k_FE_ obtained from simulations for different Poisson’s ratios, compared with analytical stiffness k_T_ from the Timoshenko solution, and the corresponding modifying parameter a(ν).

| **Poisson’s ratio** | ***k_FE_* (N/mm^3^)** | ***k_T_* (N/mm^3^)** | ***a(v)*** |
| --- | --- | --- | --- |
| 0.30 | 7.98 | 6.23 | 0.78 |
| 0.35 | 9.34 | 6.46 | 0.69 |
| 0.40 | 12.48 | 6.75 | 0.54 |
| 0.45 | 21.74 | 7.11 | 0.33 |

A modifying parameter, $a\left( v \right)=\frac{k_{T}}{k_{FE}}$ was introduced to quantify the deviation of the FE-predicted stiffness from the classical Winkler-based approximation due to lateral constraint and finite-thickness effects. The results are summarised in Table S3. Polynomial fitting of *α(v)* as a function of Poisson’s ratio shown in Figure S6c yielded the following relationship:

$$\begin{aligned} a\left( v \right)=\left( -12.50\times v^{2}+6.35\times v \right)\#\left( 1 \right) \end{aligned}$$

The fit exhibited an excellent correlation (R² = 1.0), indicating that the polynomial expression accurately reproduces the dependence of effective foundation stiffness on Poisson’s ratio. To incorporate both the Poisson-dependent term $(1-v^{2})$ from the Timoshenko solution and the fitted correction factor $a\left( v \right)$ into a single function, they were combined into a unified coefficient $\theta\left( v \right)$, expressed as:

$$\begin{aligned} \theta\left( v \right)=\frac{1}{a\left( v \right)(1-v^{2})}\#\left( 2 \right) \end{aligned}$$

Incorporating this correction into the Winkler foundation model gives the modified form:

$$\begin{aligned} k=\frac{E}{h}\times\theta\left( v \right)\#\left( 3 \right) \end{aligned}$$

This formulation provides a Poisson-ratio-dependent expression for the effective foundation stiffness, bridging the simplified Winkler model with the continuum-mechanical response of a finite-thickness elastic layer.

## S.6 Filtering noises in measured displacements

Noises may inevitably be introduced to the displacement computed by the DIC due to correlation algorithms (*2*) and the vibration of the platform. The noise was assumed to occur in all the areas of the substrate and lead to the rigid body displacements in the undeformed area of the substrate. The left edge of the substrate was assumed to be undeformed area and the displacements in X, Y and Z directions were averaged for all the subsets as below:

$$\begin{aligned} \bar{N_{x}}= \frac{\sum_{i=1}^{n} N_{ix}}{n}\# \end{aligned}$$

$$\begin{aligned} \bar{N_{y}}= \frac{\sum_{i=1}^{n} N_{iy}}{n}\#\left( 2 \right) \end{aligned}$$

$$\begin{aligned} \bar{N_{z}}= \frac{\sum_{i=1}^{n} N_{iz}}{n}\# \end{aligned}$$

where $N_{ix}$, $N_{iy}$ and $N_{iz}$ denote the computed displacement of the $i_{th}$ subsets along the left edge of the substrate in X, Y and Z direction, respectively. $n$ denotes the number of subsets along the left edge. $\bar{N_{x}}$,$\bar{N_{y}}$ and$\bar{N_{z}}$ denote to the average displacements in the X, Y and Z direction, respectively.

The noises contained in the computed displacement of subsets was filtered by subtracting the $\bar{N_{x}}$,$\bar{N_{y}}$ and$\bar{N_{z}}$ as below:

$$\begin{aligned} D_{x}= {D^{'}}_{x}-\bar{N_{x}} \# \end{aligned}$$

$$\begin{aligned} D_{y}= {D^{'}}_{y}-\bar{N_{y}}\#\left( 3 \right) \end{aligned}$$

$\begin{aligned} D_{z}= {D^{'}}_{z}-\bar{N_{z}}\# \end{aligned}$where ${D^{'}}_{x}$, ${D^{'}}_{y}$ and ${D^{'}}_{z}$ denote the computed displacements of subsets in X, Y and Z direction, respectively. $D_{x}$, $D_{y}$ and $D_{z}$ denote the displacements of subsets after filtering the noises in X, Y and Z direction, respectively. The $D_{x}$, $D_{y}$ and $D_{z}$ were used as the 3D displacement vectors which were applied on nodal points of the top surface of the PDMS substrate via FE.

As shown in Figures S7 through Figures S10, this filtering operation effectively translates the displacement fields in the X, Y, and Z directions to correct the baseline offset caused by systematic noises. This correction prevents the overestimation of displacement and traction force magnitudes. This effect is particularly evident at the boundaries of the measurement area, where the theoretical displacement and force should be zero. The plots confirm that the filtering process successfully shifts these boundary values down to near-zero levels, ensuring physical consistency.

To quantitatively evaluate the noise reduction efficacy, we analysed the residual signals at the deformation boundaries (the "remained noise") for both the control experiment and the biological application. In the steel ball case (Figures S7 and S8), the unfiltered displacement magnitude at the deformation boundary reached approximately 3.5 µm. Post-filtering, the magnitude was effectively reduced to the sub-micron level (approximately 0.5 µm). Specifically, the residual noise induced upper and lower bounds of [0.4, 1.2] µm (Figure S7b) and [0.4, 1.1] µm (Figure S8b) (within 4% of the peak displacement). Similarly, the traction force magnitude at the boundary decreased from a peak of 1.5 mN (noise-contained) to a residual level of approximately 0.2 mN, bounded by [0, 0.4] mN (within 5% of the peak load). Based on these results, the traction force uncertainty boundary is estimated to be approximately ±0.4 mN.


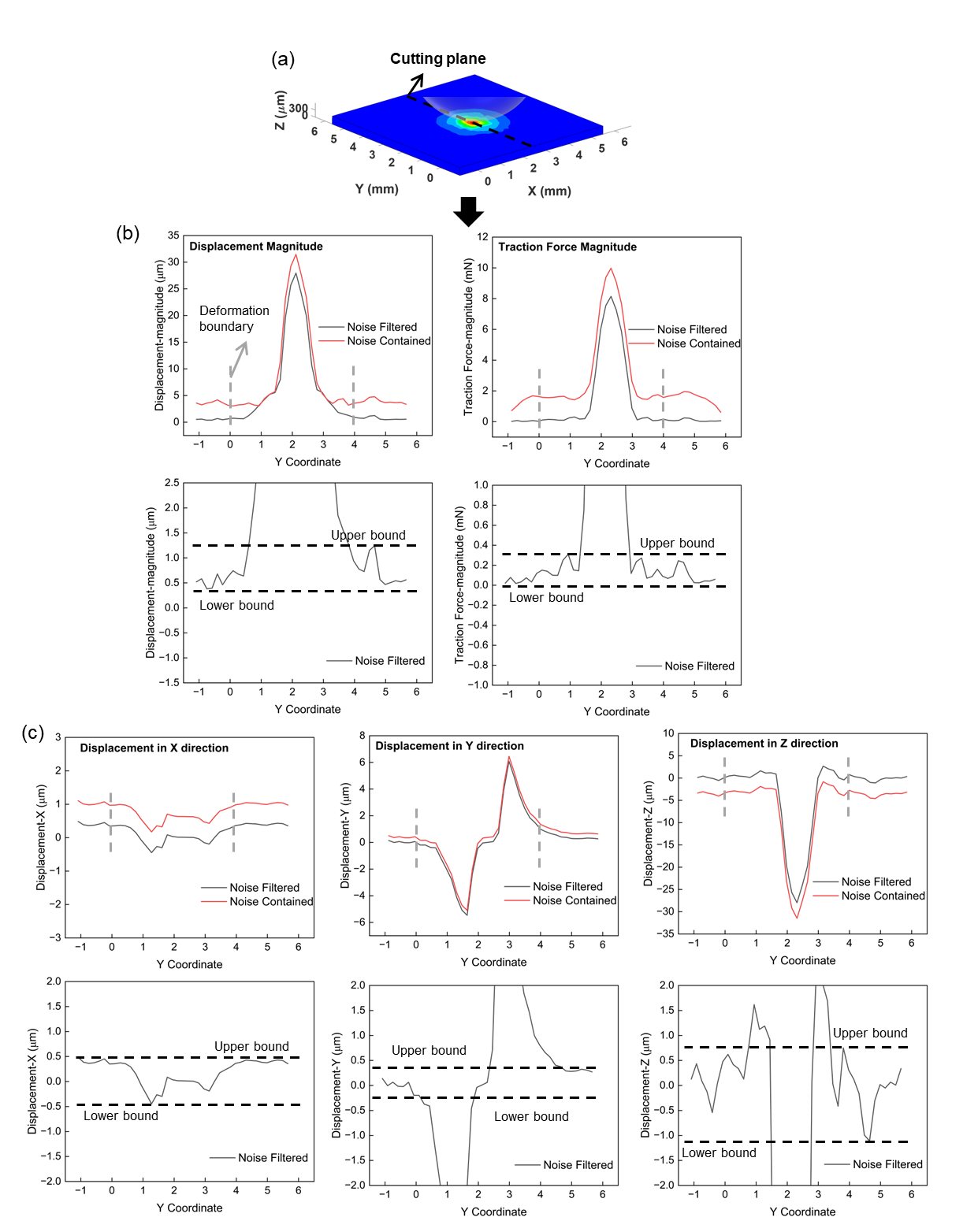


Figure S7. Steel ball under dry condition: Uncertainty quantification along the Y-axis cut. (a) Illustrative cutting view along the Y-axis at the central X-axis plane. (b) Comparison of noise-filtered and noise-contained magnitudes. The residual noise (after denoising) induces upper and lower bounds of [0.4, 1.2] µm for displacement magnitude and [0, 0.3] mN for traction force magnitude. (c) X/Y/Z displacement fields showing residual noise bounds of [-0.5, 0.5] µm, [-0.3, 0.3] µm, and [-1.1, 0.7] µm, respectively.


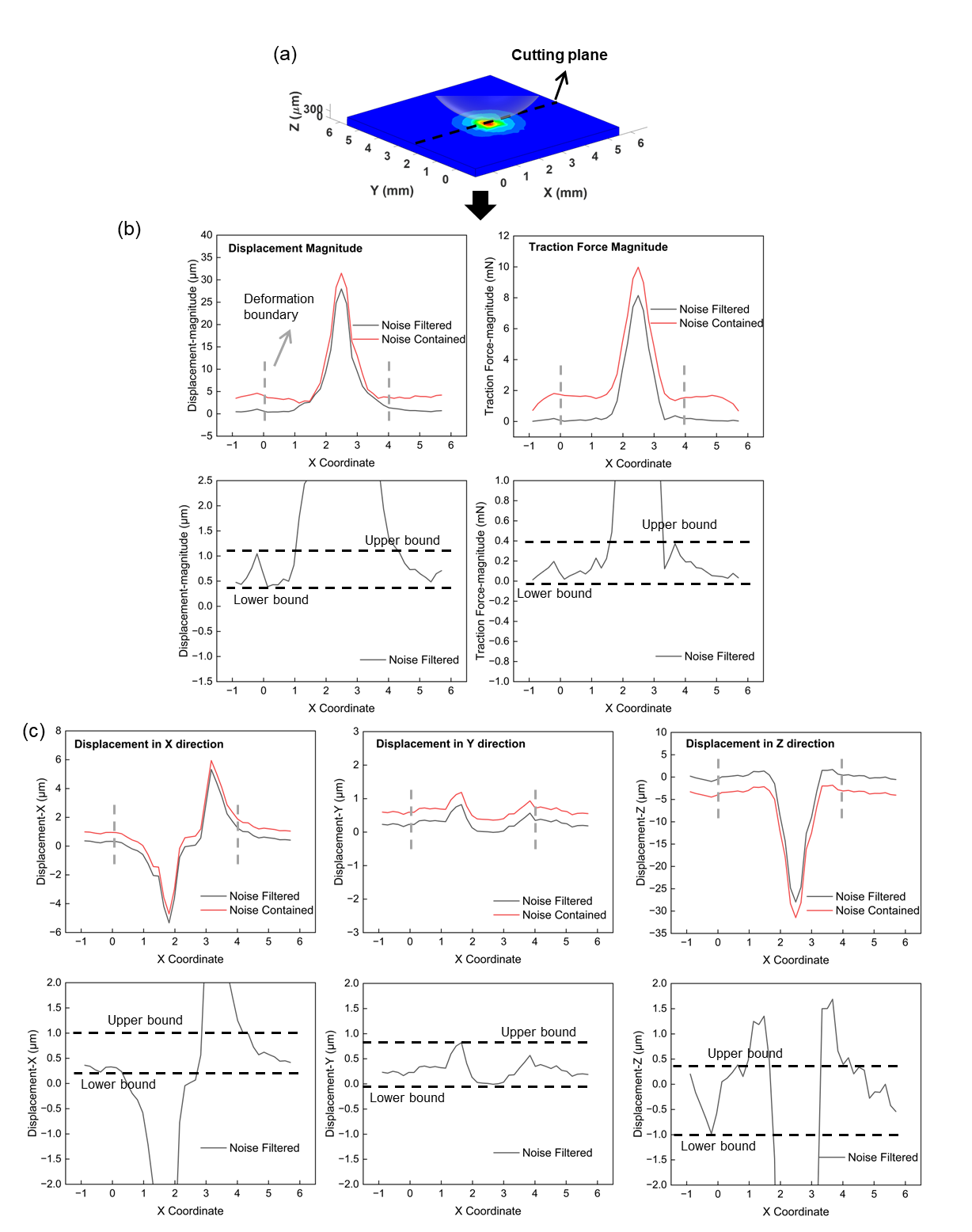


Figure S8. Steel ball under dry condition: Uncertainty quantification along the X-axis cut. (a) Illustrative cutting view along the X-axis at the central Y-axis plane. (b) Comparison of magnitudes showing residual noise induced bounds of [0.4, 1.1] µm (displacement) and [0, 0.4] mN (traction force). (c) X/Y/Z displacement fields showing residual noise bounds of [0.2, 1.0] µm, [-0.1, 0.8] µm, and [-1.0, 0.4] µm, respectively.

A similar trend was observed in the mussel plaque case (Figures S9 and S10). The displacement magnitude at the deformation boundary dropped from about 4.2 µm (noise-contained) to approximately 0.3 µm (noise-filtered). The residual noise bounds for displacement components were narrowed to generally within [-0.4, 0.4] µm (within 1% of the peak displacement). Consequently, the traction force magnitude was reduced from 1.3 mN to a residual level close to 0 mN, bounded by [0, 0.2] mN (within 5% of the peak load). Therefore, the traction force uncertainty boundary for the bio-adhesion experiments is estimated to be approximately ±0.2 mN. It was noted in the cut view along the X-axis (at the central Y-axis plane) that the noise-filtered and noise-contained traction force curves intersect at the boundary region. This suggests that while the overall systematic noise is suppressed, the traction forces at the exact periphery might still be slightly influenced by the filtering process. Future work will focus on refining the algorithm to further minimize these boundary artifacts and improve edge precision.


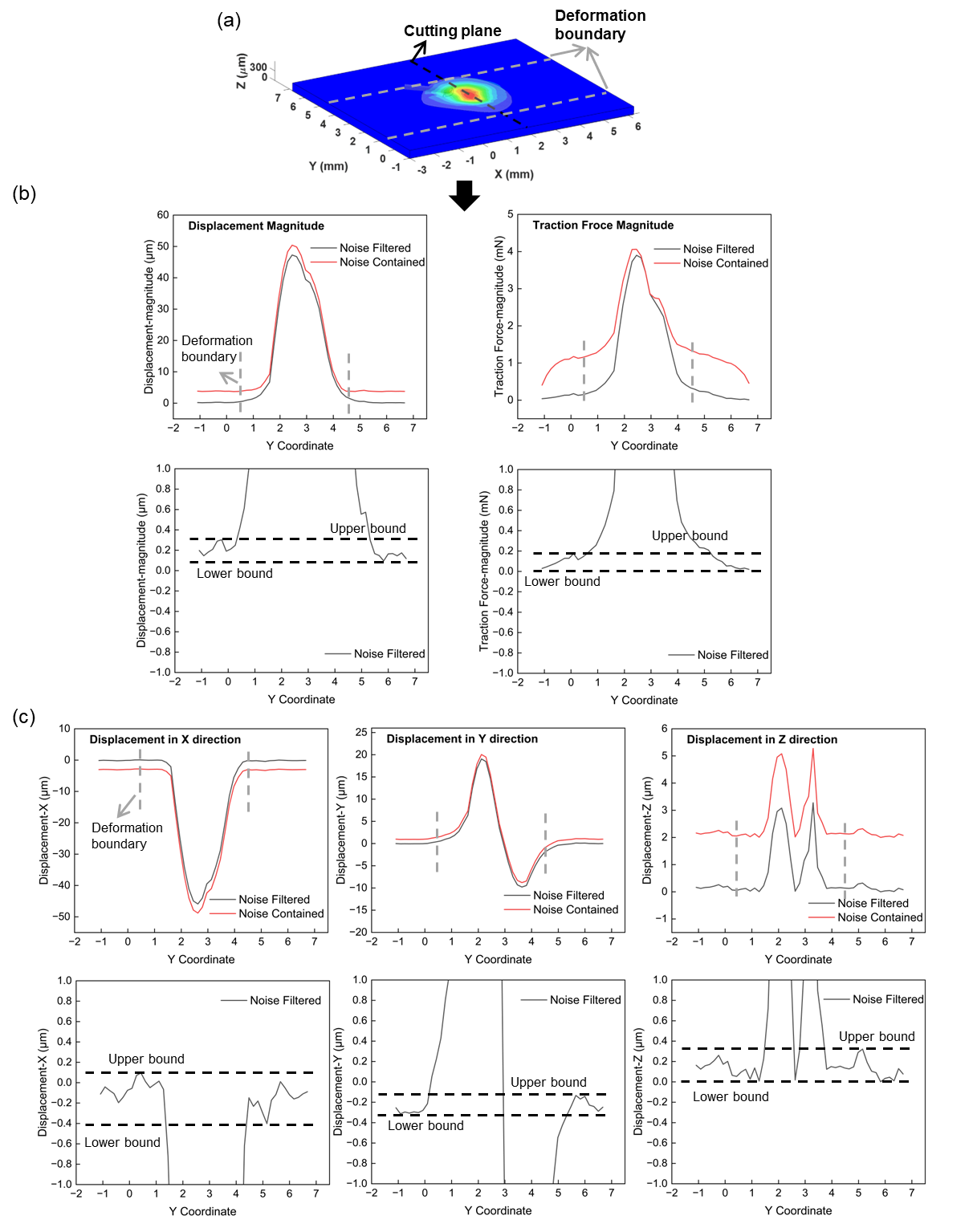


Figure S9. Mussel plaque under 15° tension: Uncertainty quantification along the Y-axis cut. (a) Illustrative cutting view along the Y-axis at the central X-axis plane. (b) Comparison of magnitudes showing residual noise induced bounds of [0.1, 0.3] µm (displacement) and [0, 0.2] mN (traction force). (c) X/Y/Z displacement fields showing residual noise bounds of [-0.4, 0.1] µm, [-0.3, -0.1] µm, and [0, 0.3] µm, respectively.


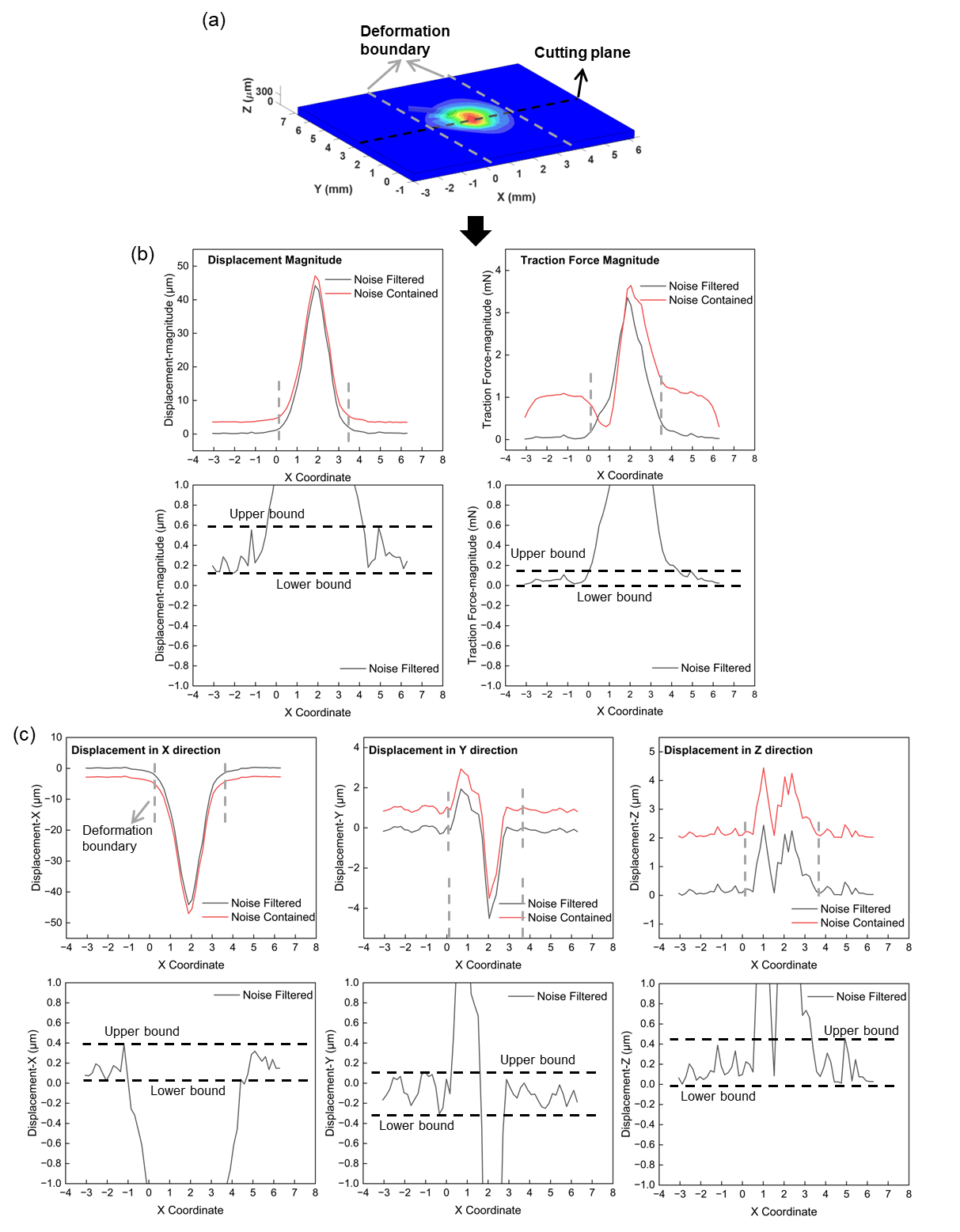


Figure S10. Mussel plaque under 15° tension: Uncertainty quantification along the X-axis cut. (a) Illustrative cutting view along the X-axis at the central Y-axis plane. (b) Comparison of magnitudes showing residual noise induced bounds of [0.1, 0.6] µm (displacement) and [0, 0.2] mN (traction force). (c) X/Y/Z displacement fields showing residual noise bounds of [0, 0.4] µm, [-0.3, 0.1] µm, and [0, 0.5] µm, respectively.

## S.7 Measurement deviations

The method was calibrated to evaluate the effects of DIC parameters on the measurement accuracy. A steel ball (diameter: 13 mm, mass: 9.0 g) was placed on the PDMS substrate in wet condition, and the substrate's deformations in the X, Y, and Z directions were measured. The results were used to calibrate the DIC parameters. The 9 g ball was selected for calibration because its gravity (~0.09 N) is close to the minimum tension force of mussel plaques’ detachment on the substate.

In current study, the numbers of subsets within the ROI region for interval $L_{i}$=10, 15, 25, 35, 45 pixels were 1748, 750, 270, 143, and 90, respectively. After determining the number (N) of subsets, the displacements of all the subsets in the X, Y, and Z directions were measured (Figure S11). To evaluate the influence of interval $L_{i}$, the mean displacement $(\overline{D_{i}})$​​ for each subset $i$ at a given interval was calculated by averaging the displacements of corresponding subsets obtained using four different subset sizes (15, 25, 35, and 45 pixels) as follows:

$$\begin{aligned} \overline{D_{i}}= {{(D}_{i1}+D_{i2}+D_{i3}+D_{i4})}/4\#\left( 5 \right) \end{aligned}$$

Here, $D_{i1}$ to $D_{i4}$​ denote the displacements of subset $i$, each obtained from displacement fields computed using the four different subset sizes, respectively. The deviation between each measured displacement ($D_{i}$) and the mean displacement was determined using the root mean square deviation (RMSD) as follows:

$$\begin{aligned} RMSD_{j}=\sqrt{\frac{\sum_{i=1}^{N} {{(D}_{i}-\overline{D_{i}})}^{2}}{N}}\#\left( 6 \right) \end{aligned}$$

where $j$ denotes the directions X, Y and Z. The deviation relative to the maximum displacement was then calculated as below:

$$\begin{aligned} \theta= \frac{RMSD_{j}}{D_{max}}\#\left( 7 \right) \end{aligned}$$

where $\theta$ is the relative deviation and $D_{max}$ is the absolute value of the maximum displacement in X, Y and Z directions. The diameter of deformed area is illustrated in Figure S11 (c), which is approximately 2.5 mm.


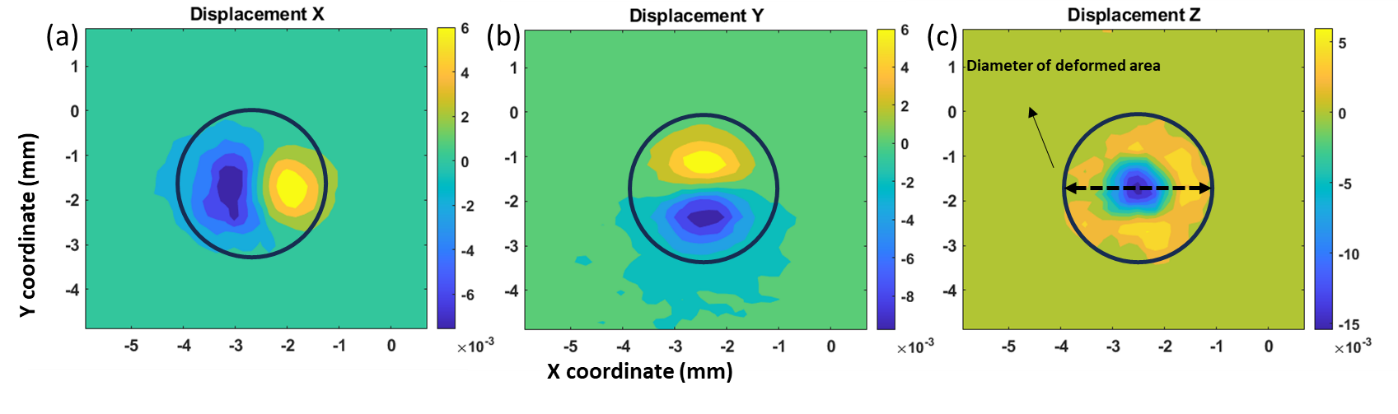


Figure S11. The measured deformations of the substrate under the 9g steel ball in X (a), Y(b) and Z(c) directions.

The distance between adjacent square subsets (x_i_, y_j_) and (x_i_, y_j-1_), was determined by calculating the absolute difference of their y-coordinates ($\left| y_{i}-y_{j-1} \right|$). Since the subsets are square, the same calculation applies to the x-coordinates. Figure S12a shows the actual distance (unit: mm) increases linearly with subset interval (unit: pixel). The slope indicates the actual length per unit pixel, i.e., 0.015 mm. Therefore, the subset intervals of 10, 15, 25, 35 and 45 pixels correspond to 0.15, 0.225, 0.375, 0.525 and 0.675 mm, respectively. Similarly, the subset sizes of 15, 25, 35, 45 pixels corresponds to 0.225, 0.375, 0.525 and 0.675 mm, respectively. The subset sizes and intervals are then normalised relative to the ROI of the substrate under 9 g steel ball as below:

$$\begin{aligned} L_{ns}= \frac{L_{s}}{d} \#\left( 3 \right) \\ L_{ni}= \frac{L_{i}}{d} \end{aligned}$$

where $L_{ns}$ and $L_{ni}$ denote the normalised subset size and interval, respectively. $d$ denotes to the above-mentioned diameter of the ROI area, i.e., 2.5 mm. Therefore, $L_{ns}$ and $L_{ni}$ are in the range of 0.06 to 0.27 and 0.09 to 20.27, respectively. The absolute values of the measured maximum displacement in X, Y and Z directions are about 6, 8, 15 µm, respectively. The $\theta$ values for the different normalized sizes and intervals are shown in Figure S12 (b-d).


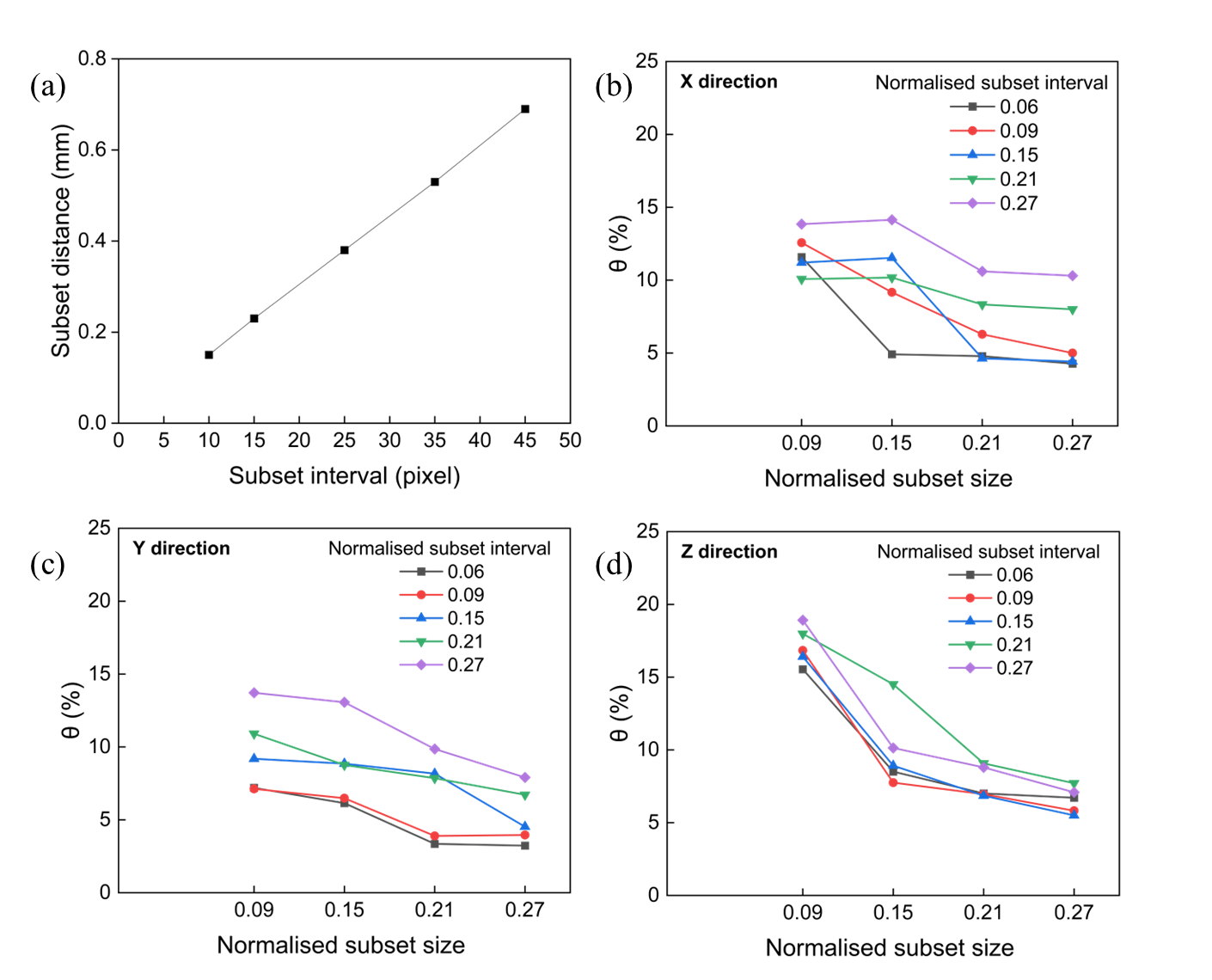


Figure S12. (a) The relationship between subset actual distance in global coordinate and the pixels in images; The deviation relative to deformation as a function of normalised size for each subset interval in X (b), Y (c) and Z (d) direction.

The results show that the displacement in Z direction has the largest $\theta$ (up to about 20%) compared to X and Y directions. The vibration on the platform is mainly vertical which leads to greater noises (up to 2 µm) in the measured Z displacement than noise (less than 1 µm) in X and Y displacements. The $\theta$ values of displacement in the three directions all decrease with the normalised sizes to the minimum of about 5%. This indicates the deviation of the measurement converges for the greater normalised size which comprises enough distinctive speckle pattern for a reliable DIC computation on the displacement.

The subset size should be kept small, as the overall displacement of the entire subset is calculated together, and smaller deformations within the subset may be missed by DIC. This drawback can be alleviated by choosing overlapped subsets when the subset interval is smaller than the subset size. The results show that the $\theta$ decreases with smaller subset intervals, indicating the measurement is more reliable for smaller subset intervals. The results suggest that the reliability of the measurements depend on the size of the subsets which should comprise enough distinctive speckles to be accurately tracked, meanwhile the interval of subsets should be small to capture the deformation of partial areas within a subset. Therefore, the largest subset size (45 pixels) and smallest subset interval (10 pixels) are used for the following tests, consistent with recommendations in the literature (*3*).

## S.8 Quantitative comparison between FE prediction and experimental measurement for steel ball indentation

To address the discrepancies between the ideal simulated contact stress fields and the experimental results, and to provide a more thorough validation beyond simple contact radius estimation, a quantitative cross-sectional analysis was conducted. Displacement fields and traction force profiles along the central axes of the contact zone for both FE simulations and experimental measurements were compared.

Figure S13 presents the comparison for the steel ball under dry conditions. First, the displacement magnitude profiles for both the experimental and FE results extend across a spatial range of 0–4 mm, effectively defining the deformation zone. The resulting deformation diameters are 3.8 mm for the experimental measurement and 4.0 mm for the simulation, respectively. Near the deformation boundary, the absolute difference of displacement magnitude between the experimental (U_Exp_) and FE (U_FE_) results was less than 3µm. While this corresponds to a maximum relative deviation, defined as |U_Exp_ - U_FE_| / U_Exp_, of approximately 33.5%, it is crucial to note that this high percentage arises because the displacement values at the boundary are approaching zero, making the ratio sensitive to small variations. This discrepancy is primarily driven by experimental noise. For instance, the experimental X displacement profile along the central Y axis exhibits wave like fluctuations compared to the smooth, linear profile of the FE prediction. In the central deformation region, where the signal to noise ratio is high, the agreement improves significantly, with the relative deviation decreasing to approximately 3.0%. A similar trend is observed in the traction force magnitude (F_Exp_ and F_FE_ refer to experimental and FE, respectively). The absolute difference decreases from less than 0.7 mN at the deformation boundary to 0.2 mN in the central region. In terms of relative accuracy, this central region demonstrates excellent agreement, corresponding to a deviation defined as |F_Exp_ - F_FE_| / F_Exp_, of approximately 1.9%.


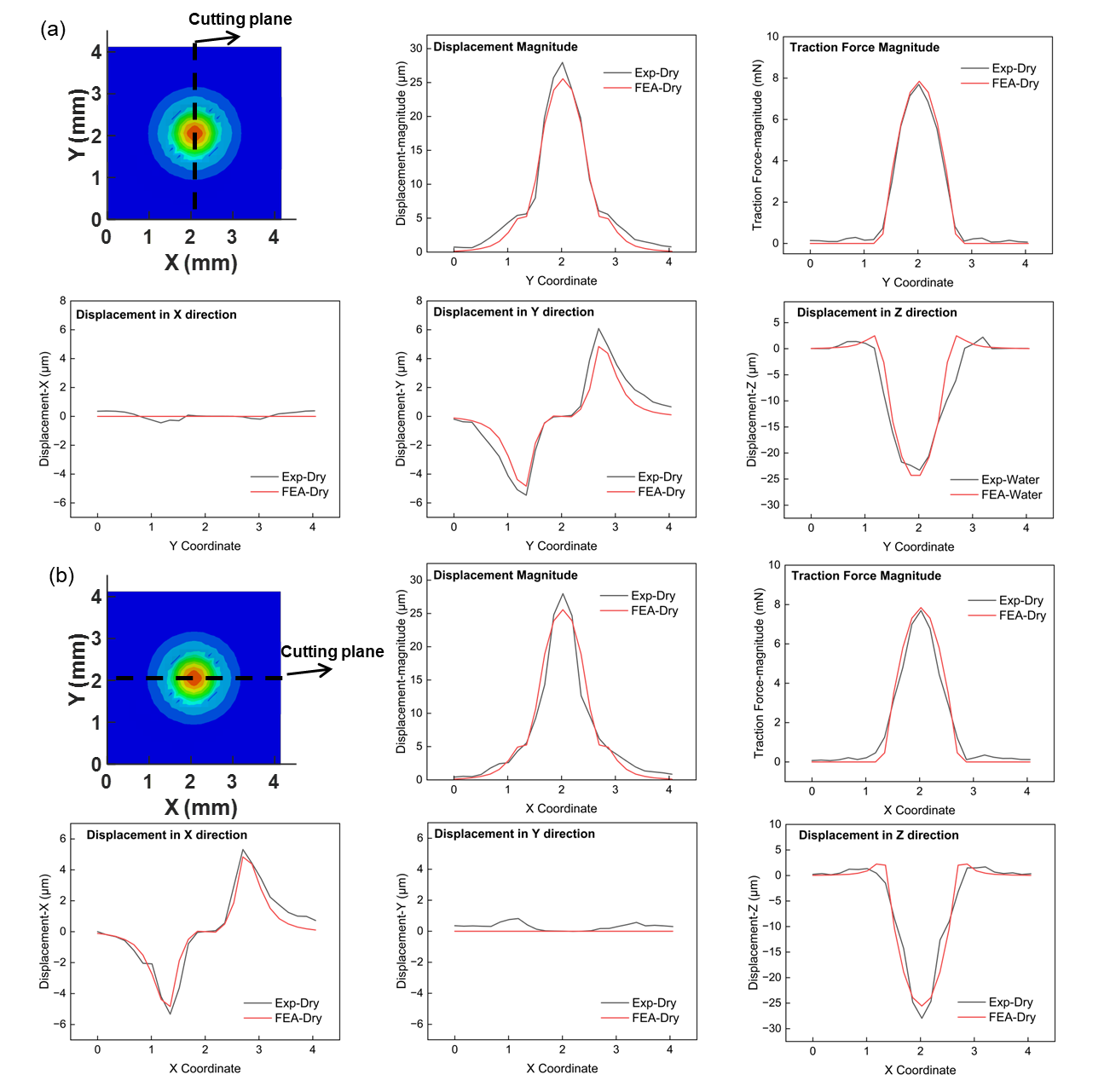


Figure S13. Steel ball under dry condition: (a) FE simulated and experimental measured displacement magnitudes, traction-force magnitudes, and X/Y/Z displacement fields shown on the cut view along the X-axis at the central Y-axis plane. (b) Corresponding fields on the cut view along the Y-axis at the central X-axis plane.

Figure S14 presents the comparison for the wet condition. The absolute difference near the deformation boundary was less than 6 µm. Although this results in a relative deviation of approximately 42.3% due to the low signal magnitude at the edge, the agreement tightens in the central region, where the relative deviation decreases to approximately 3.2%. The absolute difference in traction force magnitude ranged from less than 2.5 mN at the boundary to 0.1 mN in the central region. This central value corresponds to a relative deviation of approximately 1.2%, indicating highly accurate force reconstruction in the primary contact zone. The absolute differences at the deformation boundaries in the wet condition are slightly larger than those in the dry condition. This is attributed to the presence of water, which introduces uncertainties regarding the friction coefficient and its dynamic effect on the contact interface between the steel ball and the substrate, factors that are challenging to model perfectly in FE.


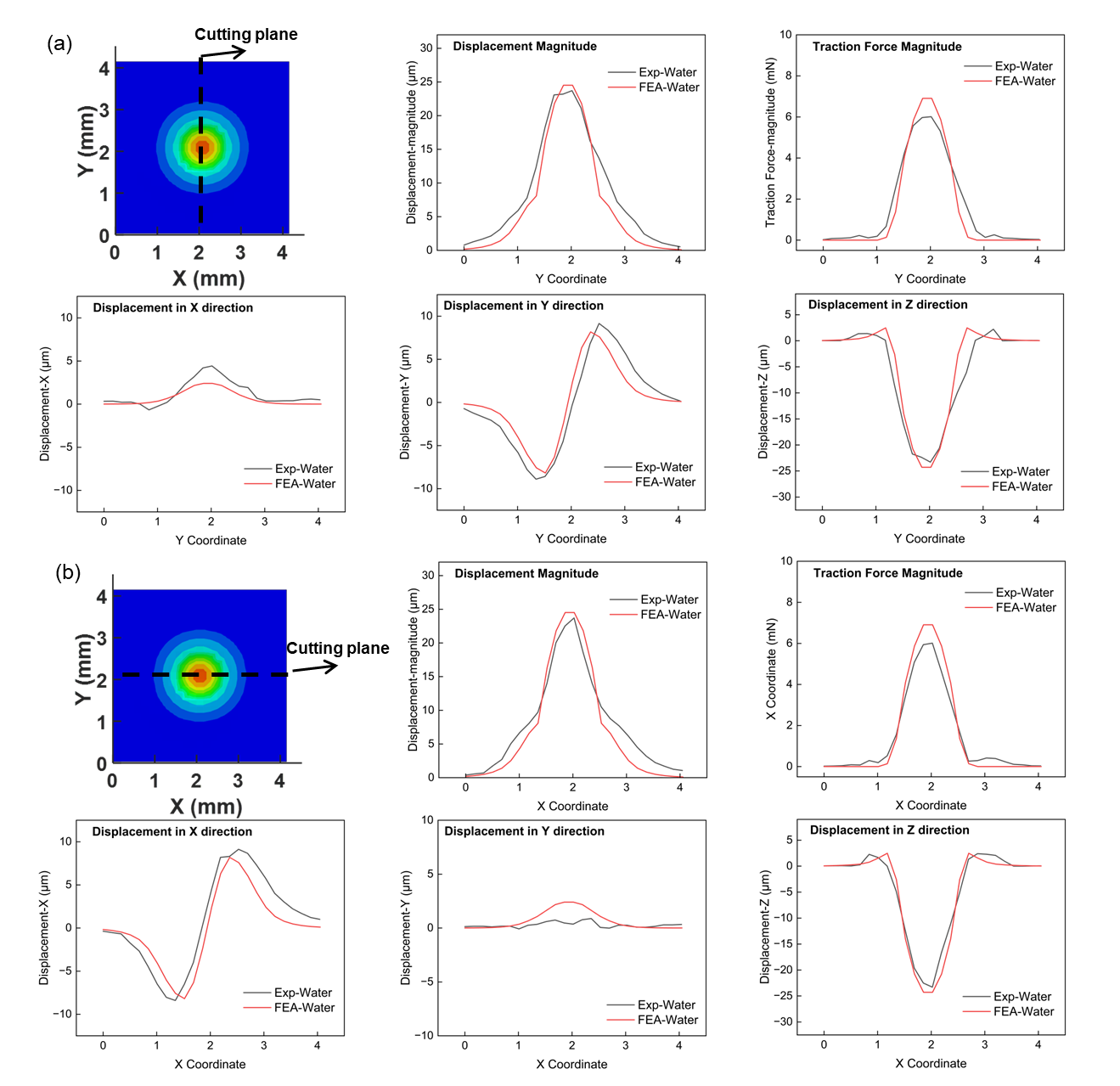


Figure S14. Steel ball under wet condition: (a) FE simulated and experimental measured displacement magnitudes, traction-force magnitudes, and X/Y/Z displacement fields shown on the cut view along the X-axis at the central Y-axis plane. (b) Corresponding fields on the cut view along the Y-axis at the central X-axis plane.

## S.9 Quantitative comparison between linear elastic and hyperelastic models in a controlled simulation

To demonstrate the theoretical necessity of hyperelastic modelling for minimizing measurement error independent of experimental noise, a direct comparison was performed using a purely numerical, well-controlled contact system.

FE simulations were conducted using a 3D substrate model, as illustrated in Figure S15a. The substrate was modeled with a thickness of 0.3 mm and a total surface area of 22.5 mm × 22.5 mm. A central region measuring 7.5 mm × 7.5 mm was subjected to a uniform surface pressure of 2 MPa in the Z-direction. The model utilized C3D8RH elements with a mesh density of approximately 475 elements/mm³, consistent with the resolution of the DIC measurements employed in the main study. The nodes at the bottom surface were fully constrained to prevent any translational or rotational movement. Two distinct material definitions were applied to the substrate: a linear elastic model (Young modulus: 1.38 MPa, Poisson’s ratio: 0.45) and a hyperelastic model based on the second-order Ogden parameters derived in Section 2.1. The Young’s modulus for the linear model was selected to correspond to the initial stiffness of the fitted Ogden model.


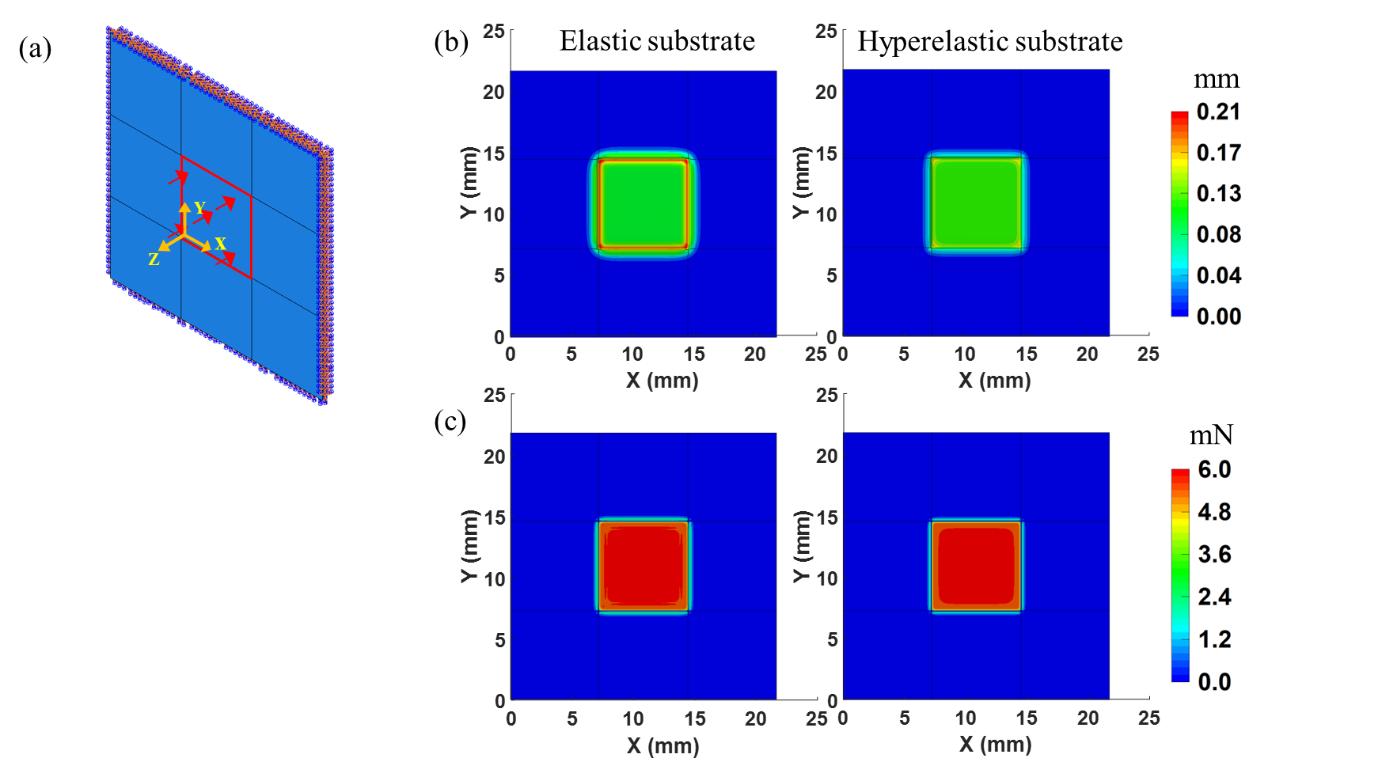


Figure S15. a) Illustration of the 3D substrate model with a central distributed load of 2 MPa; (b) Comparison of displacement magnitude profiles of linear elastic and hyperelastic models; Comparison of reaction force magnitude profiles of linear elastic and hyperelastic models

The comparison reveals that the choice of constitutive model significantly influences both the magnitude and the spatial distribution of deformation and force. As shown in Figure S15b, the displacement profiles for the two models diverge noticeably. In the central loaded region, the displacements exhibit a uniform distribution, forming a plateau with no distinct peaks. The magnitudes for the elastic and hyperelastic models are 0.095 mm and 0.107 mm, respectively, corresponding to a difference of approximately 12.6%. Critically, this deviation is not constant; at the deformation boundary, the difference increases to 19.0%, indicating that the linear model fails to accurately capture the deformation gradient. A similar location-dependent discrepancy was observed in the reaction force distribution (Figure S15c). While the difference between the models is approximately 5.7% in the central region, it widens significantly to 20.0% at the boundary of the loaded zone.

## S.10 The effect of friction between the ball and the substrate

The displacements of the friction (friction coefficient: 1.7) and frictionless models in the X, Y, and Z directions, as well as the overall displacement magnitudes, are summarized in Figure S16. The friction condition does not change circular deformation patterns. However, the diameter (approximately 2.36mm) obtained from friction model was about 7 % lower than that from the frictionless FE simulation (approximately 2.53 mm). Furthermore, the peak values of displacement of the friction model in X, Y Z directions and magnitude are 13%, 13%, 5% and 5% lower than those of the frictionless model, respectively.


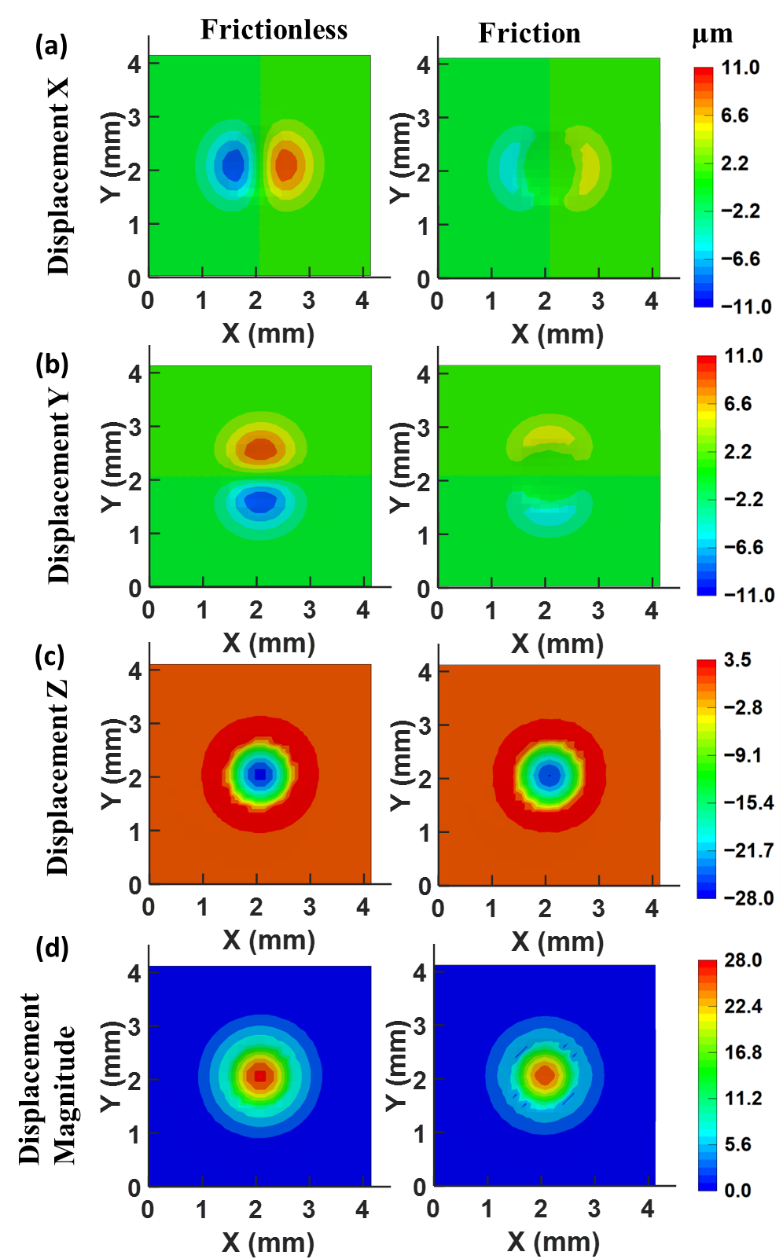


Figure S16. The FE predictions with and without friction conditions of the displacements in (a) X direction, (b) Y direction, (c) Z direction, as well as (d) displacement magnitude.

**References**

1. L. Petrone, A. Kumar, C. N. Sutanto, N. J. Patil, S. Kannan, A. Palaniappan, S. Amini, B. Zappone, C. Verma, A. Miserez, Mussel adhesion is dictated by time-regulated secretion and molecular conformation of mussel adhesive proteins. *Nat Commun* **6** (2015).
2. B. Pan, K. Qian, H. Xie, A. Asundi, Two-dimensional digital image correlation for in-plane displacement and strain measurement: A review. *Meas Sci Technol* **20** (2009).
3. Y. Su, Q. Zhang, Z. Gao, Statistical model for speckle pattern optimization. *Opt Express* **25**, 30259–30275 (2017).
